# A test of the investment model among asexual individuals: The moderating role of attachment orientation

**DOI:** 10.3389/fpsyg.2022.912978

**Published:** 2022-09-16

**Authors:** Alexandra Brozowski, Hayden Connor-Kuntz, Sanaye Lewis, Sania Sinha, Jeewon Oh, Rebekka Weidmann, Jonathan R. Weaver, William J. Chopik

**Affiliations:** Department of Psychology, Michigan State University, East Lansing, MI, United States

**Keywords:** asexuality, investment model, attachment orientation, asexual spectrum, romantic relationships

## Abstract

Many asexual individuals are in long-term satisfying romantic relationships. However, the contributors to relational commitment among asexual individuals have received little attention. How do investment model characteristics and attachment orientations predict relationship commitment among asexual individuals? Our study looked at a sample of 485 self-identified asexual individuals currently in a romantic relationship (*M_age_* = 25.61, *SD* = 6.24; *M_RelationshipLength_* = 4.42 years, *SD* = 4.74). Individuals reported on Investment Model characteristics (i.e., their relationship satisfaction, investment, alternatives, and commitment) and their attachment orientations. Satisfaction, investment, and fewer alternatives were associated with greater commitment. Attachment orientations only occasionally moderated the results: for people low in anxiety, satisfaction and investment were more strongly related to commitment compared to people high in anxiety. The current study provided an extension of the Investment Model to describe romantic relationships among asexual individuals.

## Introduction

The Investment Model (Rusbult, 1980a) posits that relationship commitment can be predicted from how happy people are in a relationship (i.e., satisfaction), how much they have invested in a relationship [i.e., investment(s)], and if there are few appealing options available to them (i.e., quality of alternatives). The Investment Model has provided a way of thinking about not only romantic relationships but, for example, also friendships (Rusbult, 1980b), organizational settings (Farrell and Rusbult, 1981), medical settings and health behavior (Putnam et al., 1994; Agnew et al., 2017), academics (Geyer et al., 1987), and athletics (Raedeke, 1997).

One untested application of the Investment Model is whether it can characterize the close relationships formed among *asexual* individuals (i.e., those with little to no sexual attraction).^1^ Asexual individuals are a sexual minority, and their members are not well studied, especially in the context of forming and maintaining romantic relationships. Understanding if the Investment Model characterizes their relationships (or not) is important for representing what predicts relational commitment, not only in heteronormative relationships, but also in all relationships that include asexual individuals. Although asexuality provides a unique test of the Investment Model and would increase their representation in the literature, it is reasonable to expect that the tenets of the Investment Model could apply to asexual relationships (Edge et al., 2021). There are likely other characteristics from the relationship literature that might help characterize asexual individuals’ relationships too. For example, an individual’s general approach toward close relationships—their attachment orientation—plays an important role in relationship commitment and functioning (Mikulincer and Shaver, 2007; Cassidy and Shaver, 2008). However, surprisingly rare are formal tests of the role attachment has in modulating the constituent pieces of the Investment Model to predict commitment (Etcheverry et al., 2013; Segal and Fraley, 2016). Will these long-established relationship frameworks apply to relationships involving asexual individuals?

### The investment model and adult attachment theory

The Investment Model conceptualizes relationship commitment as arising primarily from three factors—relationship satisfaction, quality of alternatives, and investment (Rusbult, 1980a; Rusbult et al., 1998; Le and Agnew, 2003; Tran et al., 2019). Relationship satisfaction refers to the subjective evaluation that a relationship’s positive qualities outweigh its negative qualities. When the outcomes are compared to an individual’s expectations (a comparison level), if the outcomes exceed the expectations, then individuals often report satisfaction with their relationship. Alternatives refer to the perceived desirability of alternatives to a current relationship, including the ability to have needs fulfilled from other partners, friends, family, or alone. Finally, investment refers to the resources, time, and effort put into a relationship and the lost outcomes if the relationship were terminated (Collins et al., 2011). The Investment Model is robust and wide-reaching for characterizing different types of relationships and different, often diverse, populations, including sexual/gender minorities (Barrantes et al., 2017), non-monogamous relationships (Rodrigues et al., 2019), and different romantic and non-romantic arrangements (Ledbetter et al., 2007; Flicker et al., 2021), although occasional adjustments to the measures are made (e.g., alternatives may not be measured among those in non-monogamous relationships).

An individual’s romantic attachment orientation is generally conceptualized as their position on two conceptually distinct dimensions: anxiety and avoidance (Fraley and Waller, 1998). Attachment-related anxiety reflects a preoccupation with the availability of close others (Mikulincer et al., 2002). Individuals with higher anxiety scores exhibit excessive reassurance-seeking and hypervigilance to signs of rejection and abandonment (Shaver et al., 2005). Attachment-related avoidance is characterized by chronic attempts to inhibit attachment-system activation in an effort to minimize distress expressions (Edelstein and Shaver, 2004). Individuals with higher avoidance scores generally dislike intimacy and are less likely to provide emotional support to romantic partners (Brennan et al., 1998; Li and Chan, 2012). Individuals reporting low scores on both dimensions are generally considered secure.

In addition to attachment orientations affecting interpersonal behavior (Simpson et al., 1992), an individual’s attachment orientation also affects their sense of themselves, their partners, and their relationship. For example, attachment orientations affect the attributions people make about their relationships where anxious individuals often assume the worst—ambiguous partner behavior turns into thoughtlessness or outright antagonism (Collins and Read, 1990; Collins et al., 2006). Insecure adults remember relationship interactions as more negative than they were, do not seem to benefit as much from responsive partner behaviors, and generally feel a lack of reciprocation from their partners (for anxious people) or feel smothered by their partners (for avoidant people; Edelstein and Shaver, 2004; Simpson et al., 2010; Simpson and Rholes, 2012; Chopik et al., 2014; Girme et al., 2015; Arriaga et al., 2018; Shaver and Mikulincer, 2021). Given this research then, insecure attachment orientations likely color relationships in a negative light, even when partners are responsive and relationships may be going ostensibly well. Thus, being in a happy relationship might not enhance commitment as much for insecure adults who often have doubts about their relationships. Having quality alternatives might be particularly influential for insecure adults who think their relationships are in trouble or prefer to be in another relationship or alone. Perceiving asymmetries in investment might be particularly damaging for people particularly sensitive to relationship problems. Therefore, attachment orientations might moderate Investment Model associations.

Descriptively, anxiety and avoidance are associated with lower relationship satisfaction and investment (Pistole et al., 1995). In a cross-sectional study, Etcheverry et al. (2013) found a significant direct negative association between attachment anxiety and relationship stability (i.e., anxious participants were more likely to break up). Avoidance, however, did not have this same negative association with relationship persistence but did have a negative association with relationship commitment. In both cases, Investment Model characteristics mediated these associations between attachment and relationship outcomes. In a longitudinal study, Segal and Fraley (2016) found that individuals who viewed their partner as responsive to their needs were more satisfied, invested in their relationships, and viewed alternatives to their relationships as less appealing. Highly anxious and avoidant individuals are less likely to view their partner as responsive, and moderation analyses revealed that links between Investment Model characteristics might be *stronger* for insecurely attached people. Specifically, for people who were particularly anxious or avoidant, investments and quality of alternatives more strongly influenced commitment. However, the opposite is occasionally seen with secure individuals placing more importance on investments and alternatives when evaluating commitment (Carter et al., 2013).^2^

### The investment model and attachment theory in the context of asexual individuals’ relationships

There is perhaps an unfair assumption that asexual individuals are less likely to pursue romantic relationships altogether due to their lowered interest in sexual relationships. Although asexual individuals may be less likely to pursue romantic relationships (nationally representative studies examining this question are not available), many asexual individuals indeed choose to be in romantic relationships (Robbins et al., 2016). This basic observation that people who identify as asexual enter romantic relationships has spurred qualitative and theoretical work, including how asexual individuals make sense of romantic orientations and feelings (and the absence of sexual feelings) across life (Rothblum and Brehony, 1993; DeLuzio Chasin, 2011), their engagement in non-monogamy for either their or their partners’ benefits (e.g., so their partner can pursue a sexual relationship; Scherrer, 2010), and how asexual individuals disclose their identity in social situations, including while dating (Robbins et al., 2016).

Asexuality provides a specific test of the Investment Model because relationship initiation, maintenance motivations, and desire are often unique experiences for asexual individuals and their relationships. For instance, many asexual individuals might view being non-partnered as an attractive alternative to feeling a sense of obligation to have sex with a non-asexual partner (Robbins et al., 2016). Further, at least some of the ways that people evaluate the quality of alternatives is with respect to their pursuit of sexual opportunities (Drigotas et al., 1999; Fincham and May, 2017), which might not be a consideration among asexual individuals. Therefore, it is possible that quality of alternatives might be a strong influence on commitment (by having more alternatives to a relationship) or a weak influence (because alternative sexual relationships may not be a factor). Likewise, being happy and having made investments in relationships are associated with higher commitment, and this could also be the case for asexual individuals in relationships. However, sexual activity is also considered a part of increasing interdependence that enhances commitment and investment, suggesting that shared sexual history is at least a partial component of relationship interdependence, satisfaction, and investments (Sprecher, 1998; Vanderdrift et al., 2012). Asexual individuals’ evaluation of commitment departs from non-asexual individuals’ evaluations in terms of (a) what are considered quality alternatives and (b) the uncertainty of how large a contributor sex or sexual needs are for satisfaction and investments.

Worth noting, it is likely that, regardless of the relationship configuration or sexual identities of the romantic partners/couple members, being a responsive partner is associated with good relationship outcomes (Reis et al., 2004). Additionally, being insecurely attached (e.g., being hypervigilant to a partner’s availability or being uncomfortable with emotional intimacy) is probably associated with less relationship satisfaction on average. Extending previous work that integrated adult attachment orientations and the Investment Model (Etcheverry et al., 2013; Segal and Fraley, 2016), we tested whether attachment orientations moderated the contribution of Investment Model characteristics (e.g., relationship satisfaction, investment, and quality of alternatives) in predicting commitment in self-identifying asexual individuals. The strength of association between insecure attachment and is Investment Model characteristics has varied across studies and is tested in variable, inconsistent ways (Carter et al., 2013; Segal and Fraley, 2016). Therefore, we were agnostic about the expected pattern of results.

## Materials and methods

### Participants

Participants were 485 individuals who self-identified as on the asexual spectrum and indicated “yes” to the question “Are you currently involved in a romantic relationship?” Participation was voluntary and opens to anyone who self-identified as asexual. No other screening criteria were employed; however, scores on the Asexual Identification Scale (Yule et al., 2015) suggested that nearly all participants surpassed a threshold to be considered “asexual.” This sample was part of a larger study of asexuality (Brozowski et al., 2022, Manuscript in preparation)^3^; the current report only includes asexual individuals currently in a romantic relationship; the other working paper focuses on lifespan experiences of attraction, acephobia, and well-being.

Most participants (67.0%) were recruited via Facebook groups dedicated to discussions about asexuality. The rest were recruited via asexuality-relevant subreddits on Reddit (20.2%), Tumblr (8.7%), the Asexual Visibility and Education Network (AVEN) web community forums (2.7%), and other sources (1.3%; e.g., referrals from friends). Participants ranged in age from 18 to 50 (*M_age_* = 25.61, *SD* = 6.24) and were 67.0% women, 14.8% non-binary, 8.8% men, and 9.4% other gender (i.e., third gender, self-described in another way, or preferred not to say); 14.3% of the sample identified as transgender. The race/ethnicity breakdown of the sample was 79.8% White, 4.9% Biracial, 4.9% other, 3.0% Asian, 2.8% Hispanic/Latino, and 4.6% additional race/ethnicities. In the current sample, the majority (51.6%) reported being in an exclusive dating relationship, 21.0% were married, 10.6% were engaged, 6.3% were in an open relationship, 6.0% were dating but not exclusively, and 4.5% had some other type of romantic relationship (*M_RelationshipLength_* = 4.42 years, *SD* = 4.74). Participants did not receive any compensation for participating.

Regarding romantic orientation, 28.2% identified as heteroromantic (i.e., having romantic attraction toward people of a different sex/gender), 28.0% identified as biromantic (i.e., having romantic attraction toward multiple sexes/genders), 6.7% identified as homoromantic (i.e., having romantic attraction toward people of the same sex/gender), and 6.3% identified as aromantic (i.e., not having romantic attraction toward people of any sex/gender). An additional 30.8% identified as “other” and provided their romantic orientation in an open-ended response [e.g., of those who said “other,” many respondents (50%) simply wrote “asexual” but others said demisexual (12.7%; experiencing sexual attraction after romantic attraction has been established; Carrigan, 2011), panromantic (10.6%; romantic attraction to all sexes/genders; Yule et al., 2017), grey (5.6%; wide-ranging term for those who fall in the “grey area” between sexual and asexual; Carrigan, 2011), and others that reported their orientation with less than 3% frequency for each (21.1%)].^4^ Among the three largest groups (heteroromantic, biromantic, and other), there were no significant differences in anxiety, avoidance, commitment, satisfaction, and investment (all *ps* > 0.320). Heteroromantic individuals reported lower quality of alternatives than biromantic (*p* = 0.003) and other individuals (*p* = 0.014).

This study was carried out in accordance with the recommendations of Michigan State University (MSU) Institutional Review Board (IRB# x17-448e) with informed consent being secured from all participants (documentation requirement waived but collected in accordance with the Declaration of Helsinki). A sensitivity analysis suggested that we could estimate effects as small as *f*^2^ = 0.02 at 80% power and at *α* = 0.05.

### Measures

#### The investment model scale

The Investment Model Scale was administered to measure features of relationship satisfaction, investment, quality of alternatives, and commitment (Rusbult et al., 1998). The seven-item commitment subscale (our main dependent variable; *α* = 0.85) includes items such as “I want our relationship to last forever.” The five-item relationship satisfaction subscale (*α* = 0.92) includes such items as, “My relationship is close to ideal.” The five-item investment subscale (*α* = 0.79) includes items such as “I have put a great deal into our relationship that I would lose if the relationship were to end.” The five-item quality of alternatives subscale (*α* = 0.84) includes items such as “My alternatives to our relationship are close to ideal (dating another, spending time with friends or on my own, etc.).” Participants rated the extent to which they agreed with each statement on a scale from 1 (*do not agree at all*) to 7 (*agree completely*).

#### Adult attachment orientation

We assessed adult attachment orientations with the Experiences in Close Relationships-Relationship Structures questionnaire, modified to be about close relationships/others generally (Fraley et al., 2011). The three-item *anxiety* subscale (*α* = 0.90) reflects an individual’s fear of abandonment and a hyperactivation of the attachment system. The six-item *avoidance* subscale (*α* = 0.86) reflects an individual’s discomfort with emotional and physical intimacy and chronic efforts to deactivate the attachment system. Participants rated the extent to which each statement described on a scale from 1 (*disagree strongly*) to 7 (*agree strongly*).

## Results

### Preliminary analyses

As seen in Table 1, the Investment Model characteristics were all significantly correlated with each other. Highly anxious individuals reported higher levels of commitment, higher levels of investment, and a lower quality of alternatives; but anxiety was not associated with relationship satisfaction. Highly avoidant individuals reported lower levels of relationship satisfaction, quality of alternatives, and commitment.

**Table 1:** Correlations between primary study variables.

Construct	1	2	3	4	5	*M*	*SD*
1. Commitment						6.10	1.12
2. Satisfaction	0.68^***^					5.70	1.23
3. Investment	0.48^***^	0.33^***^				4.84	1.21
4. Quality of alternatives	−0.51^***^	−0.33^***^	−0.36^***^			3.50	1.41
5. Anxiety	0.18^***^	−0.002	0.20^**^	−0.16^**^		5.15	1.58
6. Avoidance	−0.10^*^	−0.19^***^	−0.07	−0.13^**^	0.18^***^	3.62	1.24

Ns range from 483 to 485.

* *p* < 0.05;

** *p* < 0.01;

*** *p* < 0.001.

### Main results

We ran linear regressions predicting commitment from relationship satisfaction, investment, and quality of alternatives. We also controlled for age, gender (separately dummy coded each group for women [1], non-binary people [1], and those who listed another gender [1], with men serving as the reference group [0]), and transgender identity (dummy coded for transgender people [1], with non-transgender people serving as the reference group [0]). In the next step of the model, we entered attachment anxiety and avoidance and their two-way interactions with relationship satisfaction, investment, and quality of alternatives.^5^

As seen in the left panel of Table 2, commitment was associated with higher relationship satisfaction and investment and a lower quality of alternatives. The standardized regression coefficients for predicting commitment were highly comparable to what has been reported in previous meta-analyses; indeed, similar magnitudes of associations between investment model variables are often seen in the literature (Le and Agnew, 2003; Tran et al., 2019).

**Table 2:** The investment model and moderating effects of attachment.

Commitment	95% CI (*b*)							95% CI (*b*)						
	*b*	*SE*	β	*t*	*p*	LB	UB	*b*	*SE*	β	*t*	*p*	LB	UB
Intercept	5.96	0.11		52.11	<0.001	5.73	6.18	5.98	0.11		55.08	<0.001	5.76	6.19
Satisfaction	0.46	0.03	0.55	16.18	<0.001	0.40	0.52	0.46	0.03	0.55	16.34	<0.001	0.41	0.52
Investment	0.17	0.04	0.21	5.97	<0.001	0.11	0.23	0.15	0.03	0.18	5.39	<0.001	0.09	0.20
Quality of alternatives	−0.18	0.02	−0.25	−7.50	<0.001	−0.23	−0.13	−0.18	0.02	−0.26	−7.76	<0.001	−0.23	−0.14
Attachment anxiety								0.07	0.02	0.11	3.60	<0.001	0.03	0.11
Attachment avoidance								−0.02	0.03	−0.02	−0.67	0.51	−0.07	0.03
Anxiety × satisfaction								−0.05	0.02	−0.11	−3.33	0.001	−0.09	−0.02
Anxiety × investment								−0.06	0.02	−0.11	−3.43	0.001	−0.09	−0.02
Anxiety × quality of alt.								0.01	0.02	0.02	0.72	0.48	−0.02	0.04
Avoidance × satisfaction								−0.03	0.02	−0.05	−1.62	0.11	−0.07	0.01
Avoidance × investment								−0.01	0.02	−0.01	−0.35	0.73	−0.04	0.03
Avoidance × quality of alt.								−0.01	0.02	−0.02	−0.64	0.52	−0.05	0.03
Age	0.01	0.01	0.06	1.78	0.08	−0.001	0.02	0.01	0.01	0.09	2.92	0.004	0.01	0.02
Gender (Ref: Men)														
Women	0.21	0.12	0.10	1.79	0.07	−0.02	0.45	0.19	0.11	0.09	1.68	0.09	−0.03	0.41
Non-binary	−0.08	0.13	−0.03	−0.57	0.57	−0.34	0.19	−0.09	0.13	−0.03	−0.67	0.50	−0.33	0.16
Other gender	0.03	0.15	0.01	0.21	0.84	−0.26	0.32	0.11	0.14	0.03	0.77	0.44	−0.17	0.39
Transgender (Ref: no)	0.07	0.10	0.03	0.03	0.49	−0.13	0.27	0.11	0.10	0.04	1.12	0.26	−0.08	0.30

First step: *F*(8, 410) = 82.22, *p* < 0.001, *R*^2^ = 0.62. Second step: *F*(16, 402) = 50.46, *p* < 0.001, *R*^2^ = 0.67. 95% CI = 95% confidence interval. LB, lower-bound; UB, upper-bound.

**Figure 1:**
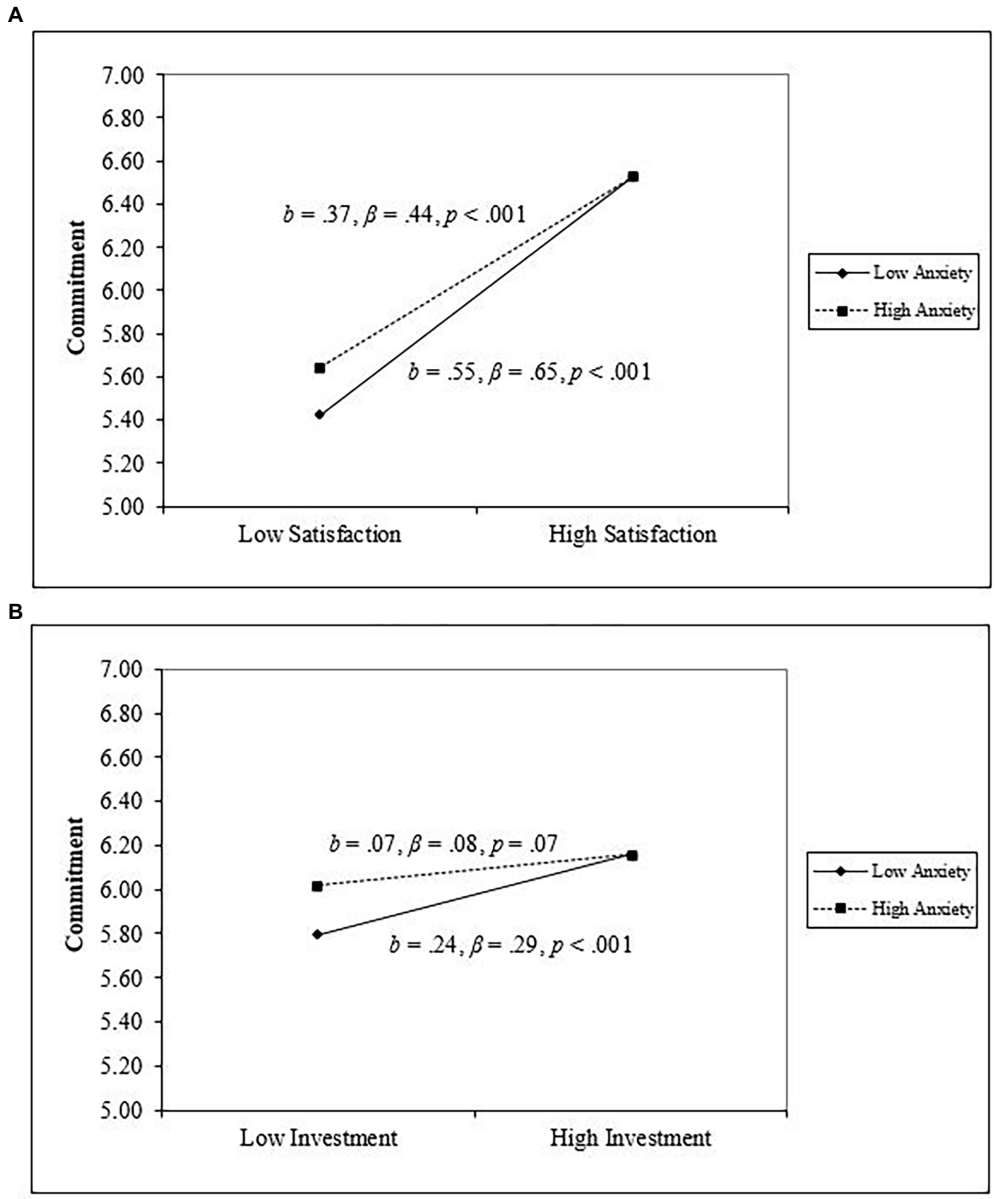
Interactions between attachment anxiety and investment model characteristics [Relationship Satisfaction **(A)**, Investment **(B)**] predicting commitment.

As seen in the right panel of Table 2, anxiety was associated with higher commitment, and avoidance was not a significant predictor of commitment. For the most part, anxiety and avoidance did not moderate the associations between Investment Model characteristics and commitment, with two exceptions: significant anxiety × relationship satisfaction and anxiety × investment interactions predicting commitment. As seen in Figure 1A, among individuals high in attachment anxiety, relationship satisfaction was positively associated with commitment (β = 0.44, *p* < 0.001). Among individuals low in attachment anxiety, relationship satisfaction was also positively associated with commitment, but to a larger degree (β = 0.65, *p* < 0.001). A similar pattern can be found in Figure 1B for the anxiety × investment interaction. Among individuals high in attachment anxiety, investment was positively associated with commitment (β = 0.08, *p* = 0.07). Among individuals low in attachment anxiety, investment was also positively associated with commitment, but to a larger degree (β = 0.29, *p* < 0.001). Altogether, relationship satisfaction and investment seem to be a stronger predictor of commitment among asexual individuals low in attachment anxiety.

## Discussion

Relationship satisfaction, quality of alternatives to a relationship, and relationship investment are thought to be antecedents of commitment. In two meta-analyses, relationship satisfaction had the largest associations (β = 0.47–0.51) with commitment, and investment and quality of alternatives had relatively comparable associations with commitment (βs = |0.19|−|0.28|). Our study was consistent with this previous work (Le and Agnew, 2003; Tran et al., 2019). Thus, we generally view the Investment Model as an appropriate theoretical framework to characterize asexual individuals’ relationships.

### The investment model and attachment theory among asexual individuals in a romantic relationship

When examining the role of attachment orientations in the context of diverse relationships (Moors et al., 2015), a few general patterns are often comparable in heteronormative samples. Namely, attachment orientations are often correlated with relationship functioning in some predictable ways. Avoidant individuals reported lower commitment, relationship satisfaction, and investment. Inconsistent with previous findings, anxiety was related to higher relationship satisfaction (instead of a null association) and more commitment (instead of less). Insecure attachment was associated with perceiving alternatives as lower quality instead of higher quality for highly avoidant people (DeWall et al., 2011) or instead of null associations for highly anxious people (Etcheverry et al., 2013). Asexual individuals low in anxiety had *stronger* associations between Investment Model characteristics and commitment compared to asexual individuals high in anxiety. It could be the case that highly anxious people might not be benefitting from the commitment-enhancing functions of higher levels of relationship satisfaction and investments; perhaps this is because of their rumination on the worst parts of their relationship and inferring the most unflattering motives of their partners (Collins and Read, 1990; Pietromonaco and Barrett, 2000; Simpson et al., 2010; Arriaga et al., 2018). Of course, closer examination of the simple slopes revealed that both individuals high and low in anxiety benefit from being in a satisfying and invested relationship, but people low in anxiety benefit more. Worth noting, there could be several reasons for these differences—although it might be attributable to particulars about living life as an asexual person, variation can also arise from variability in sampling distributions, measurement considerations, a combination of these reasons, or other reasons entirely.

### Limitations and future directions

In the current study, we relied primarily on cross-sectional data from individuals and had little information about their relational behavior with their partners. Are they dating other asexual people or non-asexual people? If they are in relationships with non-asexual people, do asexual individuals engage in some forms of physical affection or sexual activity but not others? Examining relationship dynamics among asexual individuals and how they might (not) negotiate sexual activity with non-asexual partners is a promising future direction of its own. Future research may benefit from looking at their partners’ sexual and romantic identities, their perspective on the relationship, and their relationship satisfaction. Given this context, it is possible that views toward sexual intimacy and the relationship would be *discordant*, particularly if the people in the relationship have different identities. Future research can more formally collect dyadic data to examine the perspectives of both members of the relationship. To improve on the current study’s cross-sectional design future research can utilize experimental (Tan and Agnew, 2016; Baker et al., 2017) and longitudinal (Impett et al., 2001; Brooks et al., 2018) tests of the Investment Model by testing these processes in the context of asexual individuals navigating relationship dynamics.

Finally, recruiting nationally representative data on asexual and non-asexual populations is important for drawing conclusions about the applicability of theoretical models of close relationships among different substrata of the population. In the current study, we focused on variation within a group of individuals on the asexual spectrum. Another approach would have been to recruit a comparable sample of non-asexual individuals and draw more formal comparisons rather than informal comparisons with published meta-analyses (e.g., Edge et al., 2021). Future research can be more deliberate in sampling and recruitment approaches to examine the generalizability of relationship phenomena. More generally, the current study can provide some reflection on whether our relational phenomena and measures are inclusive enough to capture the full diversity of relationship phenomena (e.g., removing the alternatives scale for non-monogamous couples; Rodrigues et al., 2019). In many cases, sex is often “baked into” many relationship measures and is often considered an antecedent of individual and relationship well-being (Muise et al., 2015). Can a relationship theory and phenomena truly characterize diverse relationships if their representative measures need to be revised before being administered to diverse populations? A definitive answer to this question is beyond the scope of the current paper. Future research should more critically examine and develop the conditions under which dominant theories of relationship phenomena can come to characterize diverse populations.

## Conclusion

The current study was the first to examine Investment Model characteristics and attachment orientation moderators among partnered asexual individuals. All Investment Model characteristics were significantly correlated with each other and predicted commitment among asexual individuals. Attachment-related differences were variable—a consideration when examining mechanisms linking individual differences to relational outcomes among asexual individuals. Overall, this study furthers our knowledge and understanding of marginalized and underrepresented community members and potentially normalizes asexual individuals’ individual and relational experiences.

## Data availability statement

The raw data supporting the conclusions of this article will be made available by the authors, without undue reservation.

## Ethics statement

The studies involving human participants were reviewed and approved by Michigan State University Institutional Review Board (MSU IRB# x17-448e). The patients/participants provided their written informed consent to participate in this study.

## Author contributions

AB, JW, and WC conceived of the study. WC, JO, and RW analyzed the data and created the tables and figures. AB, HC-K, SL, and SS drafted the initial manuscript. All authors provided critical edits. All authors contributed to the article and approved the submitted version.

## Conflict of interest

The authors declare that the research was conducted in the absence of any commercial or financial relationships that could be construed as a potential conflict of interest.

## Publisher’s note

All claims expressed in this article are solely those of the authors and do not necessarily represent those of their affiliated organizations, or those of the publisher, the editors and the reviewers. Any product that may be evaluated in this article, or claim that may be made by its manufacturer, is not guaranteed or endorsed by the publisher.

## Footnotes

^1^The asexual spectrum is a heterogeneous community with myriad identity labels that make up the spectrum (Carrigan, 2011). This study allowed for representation of this diverse community through participants self-reporting their identity labels. For the sake of simplicity, "asexual" is generally used as a blanket term for this community throughout this report and is not meant to diminish the entire spectrum or sub-labels within the spectrum.

^2^In choosing our analytic approach, we elected to run a relatively straightforward test of moderation which was surprisingly rare to find in the literature and departed a bit from the main studies we cited examining attachment and investment model characteristics. Specifically, Etcheverry et al. (2013) ran a cross-sectional mediation that examined attachment orientations as antecedents of investment. Segal and Fraley (2016) ran a multi-level SEM that examined attachment modeled as antecedents of investment. Perhaps most closely related to our study was Carter et al. (2013) who experimentally manipulated investment model characteristics to eventually run multi-group invariance tests based on a dichotomized measure of attachment. Based on the descriptive goals of the current study, we elected to test moderation by using the continuous measure of attachment in the context of linear regressions.

^3^Brozowski, A., Weidmann, R., Oh, J., Weaver, J. R., and Chopik, W. J. (2022). Demographic and experiential characteristics of asexual individuals and associations with well-being. Manuscript in preparation.

^4^Participants were not provided with definitions of romantic orientations and self-reported their romantic identities. Therefore, these romantic identity definitions may not fully represent each participant’s romantic identity conceptualization. In fact, within the community is a wide variety of ways to characterize thoughts, feelings, and behavior on the asexuality spectrum. For example, there is not a singular definition for the term panromantic (Belous and Bauman, 2017), and panromantic people may conceptualize and build their identities in a multitude of ways (Lapointe, 2017). Further, we did not conduct a thorough assessment of identities across the asexual spectrum—rather, people volunteered these labels when asked about their romantic orientation. Although the minority of participants wrote a more specific identity (e.g., demisexual), we are not sure about the distribution of asexual identities within our sample. Because of this, we encourage caution in generalizing the results of the current study to the entire asexual community.

^5^For the most part, our study replicated the findings from Etcheverry et al. (2013) with either comparable or smaller effect sizes (see supplement). However, satisfaction did not mediate the link between anxiety and commitment; also, quality of alternatives mediated attachment-commitment links but in the opposite direction, such that attachment insecurity was associated with perceiving fewer alternatives, which in turn was associated with higher commitment.

## References

[ref1] Agnew C. R. Harvey S. M. VanderDrift L. E. Warren J. (2017). Relational underpinnings of condom use: findings from the project on partner dynamics. Health Psychol. 36, 713–720. doi: 10.1037/hea0000488, PMID: 28277704 PMC5476490

[ref2] Arriaga X. B. Kumashiro M. Simpson J. A. Overall N. C. (2018). Revising working models across time: relationship situations that enhance attachment security. Personal. Soc. Psychol. Rev. 22, 71–96. doi: 10.1177/1088868317705257 28573961

[ref3] Baker L. R. McNulty J. K. VanderDrift L. E. (2017). Expectations for future relationship satisfaction: unique sources and critical implications for commitment. J. Exp. Psychol. Gen. 146, 700–721. doi: 10.1037/xge0000299, PMID: 28368196 PMC5411291

[ref4] Barrantes R. J. Eaton A. A. Veldhuis C. B. Hughes T. L. (2017). The role of minority stressors in lesbian relationship commitment and persistence over time. Psychol. Sex. Orientat. Gend. Divers. 4, 205–217. doi: 10.1037/sgd0000221, PMID: 28695154 PMC5501283

[ref5] Belous C. K. Bauman M. L. (2017). What’s in a name? Exploring pansexuality online. J. Bisex. 17, 58–72. doi: 10.1080/15299716.2016.1224212

[ref6] Brennan K. A. Clark C. L. Shaver P. R. (1998). “Self-report measurement of adult attachment: an integrative overview,” in Attachment Theory and Close Relationships. eds. Simpson J. A. Rholes W. S. (New York, NY: Guilford Press), 46–76.

[ref7] Brooks J. E. Ogolsky B. G. Monk J. K. (2018). Commitment in interracial relationships: dyadic and longitudinal tests of the investment model. J. Fam. Issues 39, 2685–2708. doi: 10.1177/0192513X18758343

[ref8] Carrigan M. (2011). There’s more to life than sex? Difference and commonality within the asexual community. Sexualities 14, 462–478. doi: 10.1177/1363460711406462

[ref9] Carter A. M. Fabrigar L. R. MacDonald T. K. Monner L. J. (2013). Investigating the interface of the investment model and adult attachment theory. Eur. J. Soc. Psychol. 43, 661–672. doi: 10.1002/ejsp.1984

[ref10] Cassidy J. Shaver P. R. (2008). Handbook of Attachment: Theory, Research, and Clinical Applications. 2nd *Edn*. New York, NY: Guilford Press.

[ref11] Chopik W. J. Edelstein R. S. van Anders S. M. Wardecker B. M. Shipman E. L. Samples-Steele C. R. (2014). Too close for comfort? Adult attachment and cuddling in romantic and parent-child relationships. Personal. Individ. Differ. 69, 212–216. doi: 10.1016/j.paid.2014.05.035

[ref12] Collins N. L. Ford M. B. Feeney B. C. (2011). “An attachment-theory perspective on social support in close relationships,” in Handbook of interpersonal psychology: Theory, research, assessment, and therapeutic intervention. eds. Horowitz L. N. Strack S. (Hoboken, NJ: Wiley), 46–76.

[ref13] Collins N. L. Ford M. B. Guichard A. C. Allard L. M. (2006). Working models of attachment and attribution processes in intimate relationships. Personal. Soc. Psychol. Bull. 32, 201–219. doi: 10.1177/0146167205280907, PMID: 16382082

[ref14] Collins N. L. Read S. J. (1990). Adult attachment, working models, and relationship quality in dating couples. J. Pers. Soc. Psychol. 58, 644–663. doi: 10.1037/0022-3514.58.4.644, PMID: 14570079

[ref15] DeLuzio Chasin C. J. (2011). Theoretical issues in the study of asexuality. Arch. Sex. Behav. 40, 713–723. doi: 10.1007/s10508-011-9757-x, PMID: 21541791

[ref16] DeWall C. N. Lambert N. M. Slotter E. B. Pond R. S. Jr. Deckman T. Finkel E. J.. (2011). So far away from one's partner, yet so close to romantic alternatives: avoidant attachment, interest in alternatives, and infidelity. J. Pers. Soc. Psychol. 101, 1302–1316. doi: 10.1037/a0025497, PMID: 21967006

[ref17] Drigotas S. M. Safstrom C. A. Gentilia T. (1999). An investment model prediction of dating infidelity. J. Pers. Soc. Psychol. 77, 509–524. doi: 10.1037/0022-3514.77.3.509

[ref18] Edelstein R. S. Shaver P. R. (2004). “Avoidant attachment: exploration of an oxymoron,” in Handbook of Closeness and Intimacy. eds. Mashek D. J. Aron A. P. (Mahwah, NJ: Lawrence Erlbaum Associates Publishers), 397–412.

[ref300] Edge J. M. Vonk J. Welling L. L. M. (2021). Asexuality and relationship investment: visible differences in relationship investment for an invisible minority. Psychol. Sexuality, 1–14. doi: 10.1080/19419899.2021.2013303

[ref19] Etcheverry P. E. Le B. Wu T.-F. Wei M. (2013). Attachment and the investment model: predictors of relationship commitment, maintenance, and persistence. Pers. Relat. 20, 546–567. doi: 10.1111/j.1475-6811.2012.01423.x

[ref20] Farrell D. Rusbult C. E. (1981). Exchange variables as predictors of job satisfaction, job commitment, and turnover: the impact of rewards, costs, alternatives, and investments. Organ. Behav. Hum. Perform. 28, 78–95. doi: 10.1016/0030-5073(81)90016-7

[ref21] Fincham F. D. May R. W. (2017). Infidelity in romantic relationships. Curr. Opin. Psychol. 13, 70–74. doi: 10.1016/j.copsyc.2016.03.008 28813298

[ref22] Flicker S. M. Sancier-Barbosa F. Moors A. C. Browne L. (2021). A closer look at relationship structures: relationship satisfaction and attachment among people who practice hierarchical and non-hierarchical polyamory. Arch. Sex. Behav. 50, 1401–1417. doi: 10.1007/s10508-020-01875-9 33956295

[ref23] Fraley R. C. Heffernan M. E. Vicary A. M. Brumbaugh C. C. (2011). The experiences in close relationships—relationship structures questionnaire: a method for assessing attachment orientations across relationships. Psychol. Assess. 23, 615–625. doi: 10.1037/a0022898, PMID: 21443364

[ref24] Fraley R. C. Waller N. G. (1998). “Adult attachment patterns: a test of the typological model,” in Attachment Theory and Close Relationships. eds. Simpson J. A. Rholes W. S. (New York, NY: Guilford Press), 77–114.

[ref25] Geyer P. D. Brannon Y. S. Shearon R. W. (1987). The prediction of students' satisfaction with community college vocational training. Aust. J. Psychol. 121, 591–597. doi: 10.1080/00223980.1987.9712688

[ref26] Girme Y. U. Overall N. C. Simpson J. A. Fletcher G. J. O. (2015). “All or nothing”: attachment avoidance and the curvilinear effects of partner support. J. Pers. Soc. Psychol. 108, 450–475. doi: 10.1037/a0038866, PMID: 25751717

[ref27] Impett E. A. Beals K. P. Peplau L. A. (2001). Testing the investment model of relationship commitment and stability in a longitudinal study of married couples. Curr. Psychol. 20, 312–326. doi: 10.1007/s12144-001-1014-3

[ref28] Lapointe A. A. (2017). “It’s not pans, it’s people”: student and teacher perspectives on bisexuality and pansexuality. J. Bisex. 17, 88–107. doi: 10.1080/15299716.2016.1196157

[ref29] Le B. Agnew C. R. (2003). Commitment and its theorized determinants: a meta-analysis of the investment model. Pers. Relat. 10, 37–57. doi: 10.1111/1475-6811.00035

[ref30] Ledbetter A. M. Griffin E. Sparks G. G. (2007). Forecasting “friends forever”: a longitudinal investigation of sustained closeness between best friends. Pers. Relat. 14, 343–350. doi: 10.1111/j.1475-6811.2007.00158.x

[ref31] Li T. Chan D. K. S. (2012). How anxious and avoidant attachment affect romantic relationship quality differently: a meta-analytic review. Eur. J. Soc. Psychol. 42, 406–419. doi: 10.1002/ejsp.1842

[ref32] Mikulincer M. Gillath O. Shaver P. R. (2002). Activation of the attachment system in adulthood: threat-related primes increase the accessibility of mental representations of attachment figures. J. Pers. Soc. Psychol. 83, 881–895. doi: 10.1037/0022-3514.83.4.881, PMID: 12374442

[ref33] Mikulincer M. Shaver P. R. (2007). Attachment in Adulthood: Structure, Dynamics, and Change. New York, NY: The Guilford Press.

[ref34] Moors A. C. Conley T. D. Edelstein R. S. Chopik W. J. (2015). Attached to monogamy? Avoidance predicts willingness to engage (but not actual engagement) in consensual non-monogamy. J. Soc. Pers. Relat. 32, 222–240. doi: 10.1177/0265407514529065

[ref35] Muise A. Schimmack U. Impett E. A. (2015). Sexual frequency predicts greater well-being, but more is not always better. Soc. Psychol. Personal. Sci. 7, 295–302. doi: 10.1177/1948550615616462

[ref36] Pietromonaco P. R. Barrett L. F. (2000). The internal working models concept: what do we really know about the self in relation to others? Rev. Gen. Psychol. 4, 155–175. doi: 10.1037/1089-2680.4.2.155

[ref37] Pistole M. C. Clark E. M. Tubbs A. L. (1995). Love relationships: attachment style and the investment model. J. Ment. Health Couns. 17, 199–209.

[ref38] Putnam D. E. Finney J. W. Barkley P. L. Bonner M. J. (1994). Enhancing commitment improves adherence to a medical regimen. J. Consult. Clin. Psychol. 62, 191–194. doi: 10.1037/0022-006X.62.1.191, PMID: 8034823

[ref39] Raedeke T. D. (1997). Is athlete burnout more than just stress? A sport commitment perspective. J. Sport Exerc. Psychol. 19, 396–417. doi: 10.1123/jsep.19.4.396

[ref40] Reis H. T. Clark M. S. Holmes J. G. (2004). “Perceived partner responsiveness as an organizing construct in the study of intimacy and closeness,” in Handbook of Closeness and Intimacy. eds. Mashek D. J. Aron A. P. (New York, NY: Routledge), 201–225.

[ref41] Robbins N. K. Low K. G. Query A. N. (2016). A qualitative exploration of the “coming out” process for asexual individuals. Arch. Sex. Behav. 45, 751–760. doi: 10.1007/s10508-015-0561-x, PMID: 26334774

[ref42] Rodrigues D. L. Lopes D. Pereira M. De Visser R. Cabaceira I. (2019). Sociosexual attitudes and quality of life in (non) monogamous relationships: the role of attraction and constraining forces among users of the second love web site. Arch. Sex. Behav. 48, 1795–1809. doi: 10.1007/s10508-018-1272-x, PMID: 30607714

[ref43] Rothblum E. D. Brehony K. A. (1993). Boston Marriages: Romantic But Asexual Relationships Among Contemporary Lesbians. Amherst, MA: University of Massachusetts Press.

[ref44] Rusbult C. E. (1980a). Commitment and satisfaction in romantic associations: a test of the investment model. J. Exp. Soc. Psychol. 16, 172–186. doi: 10.1016/0022-1031(80)90007-4

[ref45] Rusbult C. E. (1980b). Satisfaction and commitment in friendships. Represent. Res. Soc. Psychol. 11, 96–105.

[ref46] Rusbult C. E. Martz J. M. Agnew C. R. (1998). The investment model scale: measuring commitment level, satisfaction level, quality of alternatives, and investment size. Pers. Relat. 5, 357–387. doi: 10.1111/j.1475-6811.1998.tb00177.x

[ref47] Scherrer K. S. (2010). “Asexual relationships: what does asexuality have to do with polyamory?” in Understanding Non-Monogamies. eds. Barker M. Langdridge D. (New York, NY: Routledge), 166–171.

[ref48] Segal N. Fraley R. C. (2016). Broadening the investment model: an intensive longitudinal study on attachment and perceived partner responsiveness in commitment dynamics. J. Soc. Pers. Relat. 33, 581–599. doi: 10.1177/0265407515584493

[ref49] Shaver P. R. Mikulincer M. (2021). “Defining attachment relationships and attachment security from a personality-social perspective on adult attachment,” in Attachment: The Fundamental Questions. eds. Thompson R. A. Simpson J. A. Berlin L. J. (New York, NY: The Guilford Press), 39–45.

[ref50] Shaver P. R. Schachner D. A. Mikulincer M. (2005). Attachment style, excessive reassurance seeking, relationship processes, and depression. Personal. Soc. Psychol. Bull. 31, 343–359. doi: 10.1177/0146167204271709, PMID: 15657450

[ref51] Simpson J. A. Rholes W. S. (2012). “Adult attachment orientations, stress, and romantic relationships,” in Advances in Experimental Social Psychology. eds. Devine P. Plant A.. Vol. 45 (San Diego, CA: Academic Press), 279–328.

[ref52] Simpson J. A. Rholes W. S. Nelligan J. S. (1992). Support seeking and support giving within couples in an anxiety-provoking situation: the role of attachment styles. J. Pers. Soc. Psychol. 62, 434–446. doi: 10.1037/0022-3514.62.3.434

[ref53] Simpson J. A. Rholes W. S. Winterheld H. A. (2010). Attachment working models twist memories of relationship events. Psychol. Sci. 21, 252–259. doi: 10.1177/0956797609357175, PMID: 20424054

[ref54] Sprecher S. (1998). Social exchange theories and sexuality. J. Sex Res. 35, 32–43. doi: 10.1080/00224499809551915

[ref55] Tan K. Agnew C. R. (2016). Ease of retrieval effects on relationship commitment: the role of future plans. Personal. Soc. Psychol. Bull. 42, 161–171. doi: 10.1177/0146167215617201, PMID: 26588936

[ref56] Tran P. Judge M. Kashima Y. (2019). Commitment in relationships: an updated meta-analysis of the investment model. Pers. Relat. 26, 158–180. doi: 10.1111/pere.12268

[ref57] Vanderdrift L. E. Lehmiller J. J. Kelly J. R. (2012). Commitment in friends with benefits relationships: implications for relational and safe-sex outcomes. Pers. Relat. 19, 1–13. doi: 10.1111/j.1475-6811.2010.01324.x

[ref400] Yule M. A. Brotto L. A. Gorzalka B. B. (2015). A validated measure of no sexual attraction: The Asexuality Identification Scale. Psychol. Assess. 27, 148–160. doi: 10.1037/a0038196, PMID: 25383584

[ref58] Yule M. Brotto L. Gorzalka B. (2017). Human asexuality: what do we know about a lack of sexual attraction? Curr. Sex. Health Rep. 9, 50–56. doi: 10.1007/s11930-017-0100-y

