# Supplementary material for: A test of the investment model among asexual individuals: The moderating role of attachment orientation
Source: Front Psychol. 2022 Sep 16;13:912978. doi: 10.3389/fpsyg.2022.912978 (PMC9523605; doi:10.3389/fpsyg.2022.912978)
Supplement: Supplementary file 1 [file Data_Sheet_1.docx]

**Supplemental Material**

As supplemental analyses, we aimed to reproduce the results reported by Etcheverry et al. (2013) who tested whether satisfaction, quality of alternatives, and investment mediated the link between attachment orientations and commitment. To that aim, we conducted a path mediation model using Mplus (Muthén & Muthén, 2017). Supplemental Figure 1 shows the path mediation model with standardized regression coefficients (the complete results can be found in Supplemental Table 1). Supplemental Table 2 shows the indirect effects of the mediations. In every but one case, satisfaction, quality of alternatives, and investment significantly mediated the associations between anxiety and commitment and avoidance and commitment. The only exception was that satisfaction did not significantly mediate the association between anxiety and commitment, which is likely due to the non-significant association between anxiety and satisfaction.

**Supplemental Figure 1**

*Mediational Path Model*

*Note.* Regression coefficients are standardized.

| **Supplemental Table 1**  *Regression Estimates of Satisfaction, Investment, and Quality of Alternatives in Supplementary Figure 1* | | | | | | | |
| --- | --- | --- | --- | --- | --- | --- | --- |
|  |  |  |  |  |  | 95% Confidence Interval (*b*) | |
|  | *b* | *SE* | β | *t* | *p* | *LB* | *UB* |
| Commitment |  |  |  |  |  |  |  |
| Satisfaction | .43 | .04 | .48 | 11.50 | < .001 | .35 | .51 |
| Investment | .26 | .04 | .25 | 6.45 | < .001 | .18 | .33 |
| Quality of alternatives | -.17 | .02 | -.24 | -7.94 | < .001 | -.22 | -.13 |
| Attachment anxiety | .07 | .02 | .10 | 3.41 | .001 | .03 | .10 |
| Attachment avoidance | -.02 | .03 | -.03 | -0.88 | .38 | -.08 | .03 |
| Satisfaction |  |  |  |  |  |  |  |
| Attachment anxiety | .02 | .04 | .03 | 0.62 | .53 | -.05 | .08 |
| Attachment avoidance | -.19 | .05 | -.20 | -3.94 | < .001 | -.29 | -.10 |
| Investment |  |  |  |  |  |  |  |
| Attachment anxiety | .12 | .03 | .19 | 3.87 | < .001 | .05 | .17 |
| Attachment avoidance | -.13 | .04 | -.17 | -3.49 | < .001 | -.21 | -.06 |
| Quality of alternatives |  |  |  |  |  |  |  |
| Attachment anxiety | -.15 | .04 | -.17 | -3.68 | < .001 | -.24 | -.07 |
| Attachment avoidance | -.16 | .05 | -.14 | -3.09 | .002 | -.25 | -.05 |
| *Note.* Uncontrolled results are reported. Controlling for age, gender identity, and transgender identity did not alter the strength, direction, and significance of the results. For commitment, *R*^2^ = .61, *p* < .001; for satisfaction, *R*^2^ = .04, *p* = .04, for investment, *R*^2^ = .05, *p* = .02; and for quality of alternatives, *R*^2^ = .06, *p* = .006. | | | | | | | |

| **Supplemental Table 2**  *Indirect Effects Between Attachment Anxiety and Avoidance, Satisfaction, Investment, and Quality of Alternatives, and Commitment* | | | | | | | |
| --- | --- | --- | --- | --- | --- | --- | --- |
|  |  |  |  |  |  | 95% Confidence Interval (*b*) | |
|  | *b* | *SE* | β | *t* | *p* | *LB* | *UB* |
| Anxiety – satisfaction - commitment | .01 | .02 | .02 | .61 | .54 | -.02 | .04 |
| Avoidance – satisfaction - commitment | -.08 | .02 | -.10 | -3.73 | < .001 | -.13 | -.04 |
| Anxiety – investment - commitment | .03 | .01 | .05 | 3.21 | .001 | .01 | .05 |
| Avoidance – investment - commitment | -.03 | .01 | -.04 | -3.27 | .001 | -.06 | -.02 |
| Anxiety – quality - commitment | .03 | .01 | .04 | 3.36 | .001 | .01 | .05 |
| Avoidance – quality - commitment | .03 | .01 | .03 | 2.91 | .004 | .01 | .05 |
|  | | | | | | | |

References

Etcheverry, P. E., Le, B., Wu, T.-F., & Wei, M. (2013). Attachment and the investment model: Predictors of relationship commitment, maintenance, and persistence. *Personal Relationships, 20*, 546-567.

Muthén, L. K., & Muthén, B. O. (2017). *MPlus version 8 user's guide*. Muthen & Muthen.
